# Supplementary material for: Causal Relationship between Aging and Anorexia Nervosa: A White-Matter-Microstructure-Mediated Mendelian Randomization Analysis
Source: Biomedicines. 2024 Aug 16;12(8):1874. doi: 10.3390/biomedicines12081874 (PMC11351342; doi:10.3390/biomedicines12081874)
Supplement: Supplementary file 1 [file biomedicines-12-01874-s001.zip › Supplementary Figure/Figure S4/Singe SNP plot of MR analysis for TL to ubm-b-1586.pdf]

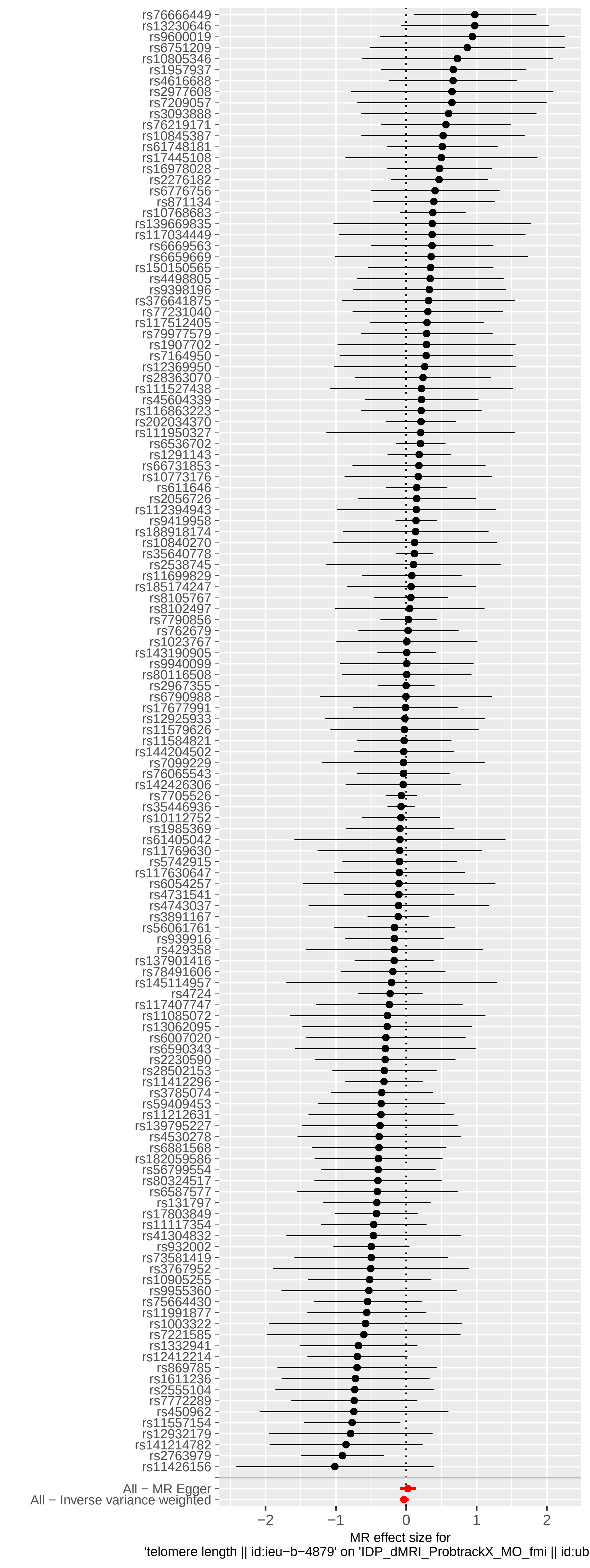
rs76666449
rs13230646
rs9600019
rs6751209
rs10805346
rs1957937
rs4616688
rs2977608
rs7209057
rs3093888
rs76219171
rs10845387
rs61748181
rs17445108
rs16978028
rs2276182
rs6776756
rs871134
rs10768683
rs139669835
rs117034449
rs6669563
rs6659669
rs150150565
rs4498805
rs9398196
rs376641875
rs77231040
rs117512405
rs79977579
rs1907702
rs7164950
rs12369950
rs28363070
rs111527438
rs45604339
rs116863223
rs202034370
rs111950327
rs6536702
rs1291143
rs66731853
rs10773176
rs611646
rs2056726
rs112394943
rs9419958
rs188918174
rs10840270
rs35640778
rs2538745
rs11699829
rs185174247
rs8105767
rs8102497
rs7790856
rs762679
rs1023767
rs143190905
rs9940099
rs80116508
rs2967355
rs6790988
rs17677991
rs12925933
rs11579626
rs11584821
rs144204502
rs7099229
rs76065543
rs142426306
rs7705526
rs35446936
rs10112752
rs1985369
rs61405042
rs11769630
rs5742915
rs117630647
rs6054257
rs4731541
rs4743037
rs3891167
rs56061761
rs939916
rs429358
rs137901416
rs78491606
rs145114957
rs4724
rs117407747
rs11085072
rs13062095
rs6007020
rs6590343
rs2230590
rs28502153
rs11412296
rs3785074
rs59409453
rs11212631
rs139795227
rs4530278
rs6881568
rs182059586
rs56799554
rs80324517
rs6587577
rs131797
rs17803849
rs11117354
rs41304832
rs932002
rs73581419
rs3767952
rs10905255
rs9955360
rs75664430
rs11991877
rs1003322
rs7221585
rs1332941
rs12412214
rs869785
rs1611236
rs2555104
rs7772289
rs450962
rs11557154
rs12932179
rs141214782
rs2763979
rs11426156
All − MR Egger
All − Inverse variance weighted
−2
−1
0
1
2
MR effect size for
'telomere length || id:ieu−b−4879' on 'IDP_dMRI_ProbtrackX_MO_fmi || id:ub
